# Evaluation of artifacts of cochlear implant electrodes in cone beam computed tomography

**DOI:** 10.1007/s00405-020-06198-y

**Published:** 2020-07-15

**Authors:** Nicholas Bevis, Thomas Effertz, Dirk Beutner, Christian Gueldner

**Affiliations:** 1 Department of Otolaryngology, University of Goettingen, Robert-Koch-Straße 40, 37075 Goettingen, Germany; 2 Department of Otolaryngology, University of Marburg, 35043 Marburg, Germany; 3 Department of Otolaryngology, Chemnitz Hospital, 09116 Chemnitz, Germany

**Keywords:** Cochlear implant, CBCT, CT, Dose reduction, Electrode artifact, Radiation dose

## Abstract

**Purpose:**

Cone Beam Computed Tomography (CBCT) offers a valid alternative to conventional Computed Tomography (CT). A possible radiation dose reduction with the use of CBCT in postoperative imaging of CIs is of great importance. Whether the visualization of Cochlear Implant (CI) electrodes in CBCT correlates with the radiation dose applied was investigated in this study.

**Methods:**

We compared the visualization quality of Contour Advance CIs to Straight CIs from Cochlear using CBCT with varying tube parameters on whole-head specimen.

**Results:**

The internal diameter of the cochlea decreases from base to apex, resulting in a significantly different intracochlear positioning of the two tested CI models. While electrodes of the Contour Advance series are located close to the modiolus, thus closer to the spiral ganglion neurons, those of the Straight series are located further away. The artifact portion of the electrode amounts to 50–70% of the radiological diameter of the electrode. An increase in artifact portion from the base (electrode #1 approx. 50%) to the apex (electrode #20 approx. 70%) of the cochlea was observed. The visualization of electrodes in the medial and apical part of the cochlea is limited due to artifact overlapping. There was no correlation between the artifact size and the applied radiation dose.

**Conclusion:**

The results indicate that a reduction of the radiation dose by up to 45% of the currently applied radiation dose of standard protocols would be possible. Investigations of the effects on subjective image quality still need to be performed.

## Introduction

With the development of the Cochlear Implant (CI), it has become possible to restore hearing in patients by substituting a non-functioning sensory epithelium (Organ of Corti) with an artificial, electrical stimulation. Current guidelines advocate for intraoperative or postoperative imaging to confirm the correct position of the electrode array in the cochlea and to detect possible misplacements or scale jumps [1]. This can lead to fibrosis and ossification of the cochlea after insertion trauma [2]. Mitigating insertion trauma is especially crucial in patients with residual hearing [3]. Intra- and postoperative imaging helps to identify possible trauma and gives the possibility for immediate revision [4]. Additionally, studies recommend postoperative imaging even after adult implantation [5]. Imaging plays an important role in the development and improvement of CIs by comparing the position of the electrodes with the achieved hearing performance and determining an optimized localization of the electrode [6, 7]. In addition to imaging, intraoperative measuring of stapedius reflex threshold values indicate the correct placement of the electrodes [8]. Cone Beam Computed Tomography (CBCT) offers a low-radiation and cost-effective alternative to conventional CT, while also being superior in visualizing high-density structures, such as the temporal bone [9]. CBCT provides a reduction of the effective dose of up to 40% of a 64-slice CT [10] and comparable radiation dose to a 128-slice CT, but with higher image quality compared to both due to the higher spatial resolution [11, 13]. The advantages in the visualization of CI electrodes result from their low susceptibility to metallic artifacts [14].

The aim of the study is to evaluate at which radiation dose a sufficient image quality can be guaranteed and whether a dose reduction in intra- and postoperative diagnostics can be achieved in everyday clinical practice.

## Methods

**Fig. 1:**
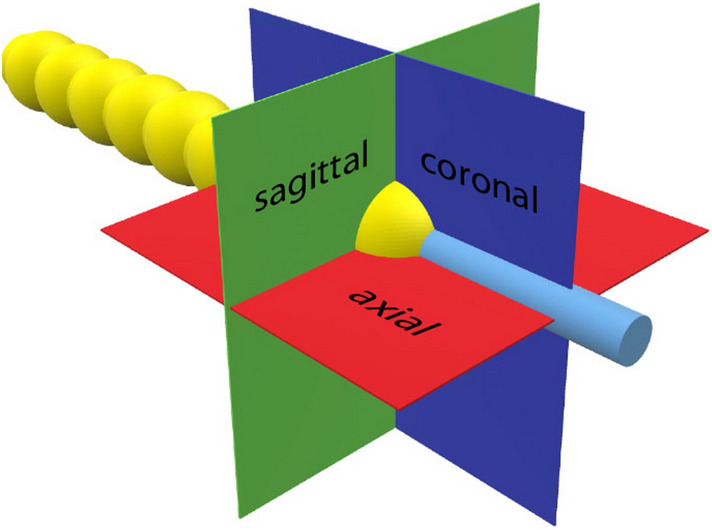
Display of the orthogonally projected planes on electrode 1

A total of four whole-head preparations of body donors were inserted with one of the four CIs of Cochlear in the left temporal bone. The IRB approval was obtained by the local ethics committee of the University of Marburg. The CI models 422 and 522 (further abbreviated with Straight) feature straight arrays, while CI models 24RE and 512 (further abbreviated with Contour) feature a pre-bent electrode array. The pre-bent electrode returns to a predefined, non-straight configuration to achieve a closer proximity to the modiolus of the cochlea. After implantation, a CBCT device by Morita (Accu-I-tomo F17, Morita, Kyoto, Japan) visualized the implant electrodes. 152 CBCT datasets were recorded by varying tube parameters, such as voltage (80–90 kV), current (4–10 mA), rotation angle (180° and 360°), and reconstruction mode. Applied radiation dosage varied in relation to exposure time, current, voltage, and program type. It ranged from 1.75 to 16 mGy and was expressed as Computed Tomography Dose Index (CTDI), which was calculated by the CBCT device. The calculation of the CTDI runs according to the manufacturer-specific protocols and is output via the device. A separate measurement has not been performed. Each one of the 152 datasets was then exported to a graphical image processing program and the images were displayed in the coronal, sagittal, and axial planes. The designated planes are positioned orthogonally on the respective electrode (Fig. 1).

The radiological diameters of electrodes #1, #11, #20, and the corresponding internal diameter of the cochlea were measured 152 times in total. The intracochlear position of the electrode was determined by measuring the distance of the boundaries of the artifact of the electrode to the outer and inner (modiolus) cochlear wall. The radiologically determined diameters were then compared with the real dimensions to calculate the corresponding artifact portion.

**Fig. 2:**
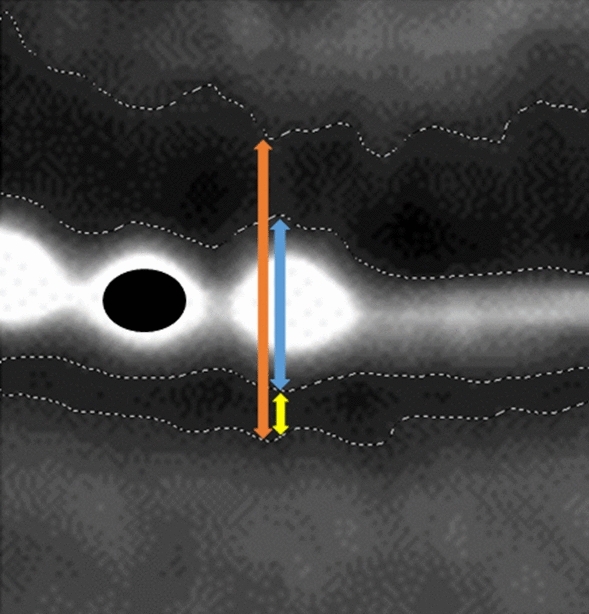
Method of measuring the artifact dimensions of electrode #1 and the surrounding structures. Blue: height of electrode; Orange: Internal diameter of the cochlea; Yellow: Distance to the outer/lateral wall of the cochlea; Black: Real diameter of electrode 2, compared to its observed artifact. Boundaries of the objects are displayed by grayscale analysis of the Image Manipulation program

The measurements were carried out using GNU Image manipulation program (GIMP 2.8). Using grayscale recognition, a consistent measurement of the boundaries of the investigated structures could be ensured. One person carried out all measurements, while the continuity was ensured by the histographical grayscale analysis of the images by the GNU Image manipulation program. Various studies in other areas describe the use of analysis tools in image processing programs as helpful and consider them equivalent to conventional manual measurement [15, 16] (Fig. 2).

Results were analyzed using Microsoft Excel 2016 and GraphPad Prism 7. Graphs were presented as mean values or single data points and the bars depict standard deviation. Unless otherwise indicated, analysis was done on four individual specimen with 38 radiological measurements each. Data were analyzed by two-way ANOVA with Sidak's multiple comparisons test, or by unpaired *t* test assuming normal distribution. A simple linear regression test was performed. A *p* value of at least < 0.05 was considered as significant.

## Results

**Fig. 3:**
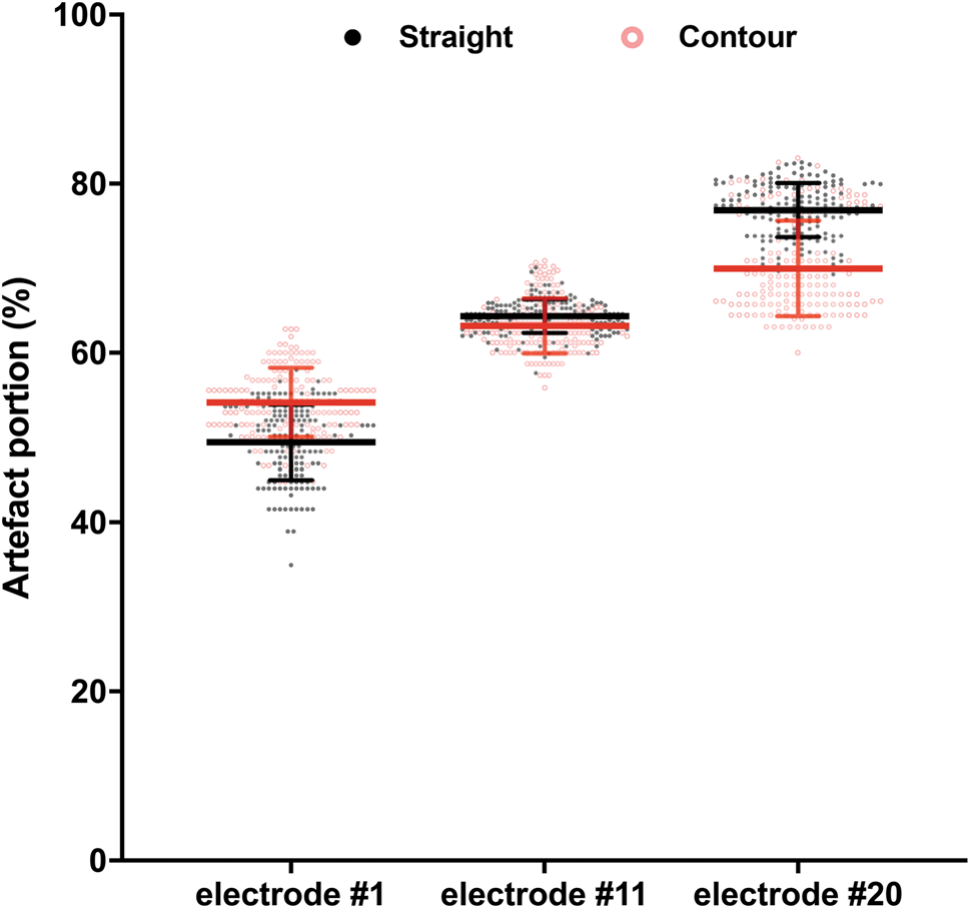
Comparison of the artifact portion of the measured radiological diameter. Difference to the mean Contour/Straight at electrode #1: 4.7%; electrode #11: 1.2%; electrode #20 – 1.2% (*p* #1 < 0.001; *p* #11 < 0.031; *p* #20 < 0.001; ANOVA two way)

The ratio of artifact to real diameter of the electrode was determined at electrodes #1, #11 and #20. The artifact portion of all measured electrode arrays was 51.8 ± 4.9% at electrode #1, 63.8 ± 2.8% at electrode #11, and 73.42 ± 5.74% at electrode #20. The artifact portion significantly increased toward the apical part of the cochlea. For electrode #1, the artifact portion of the Contour array was 4.7% greater than the portion of the Straight array. At electrode #11, the artifact portion of the Straight array was 1.2% greater than the artifact portion of the Contour array until it ultimately increased to 6.9% at electrode #20 (electrode #1 *p* < 0.001;electrode #11 *p* < 0.031; electrode #20 *p* < 0.001; ANOVA two way, Straight vs. Contour) (Fig. 3).

**Fig. 4:**
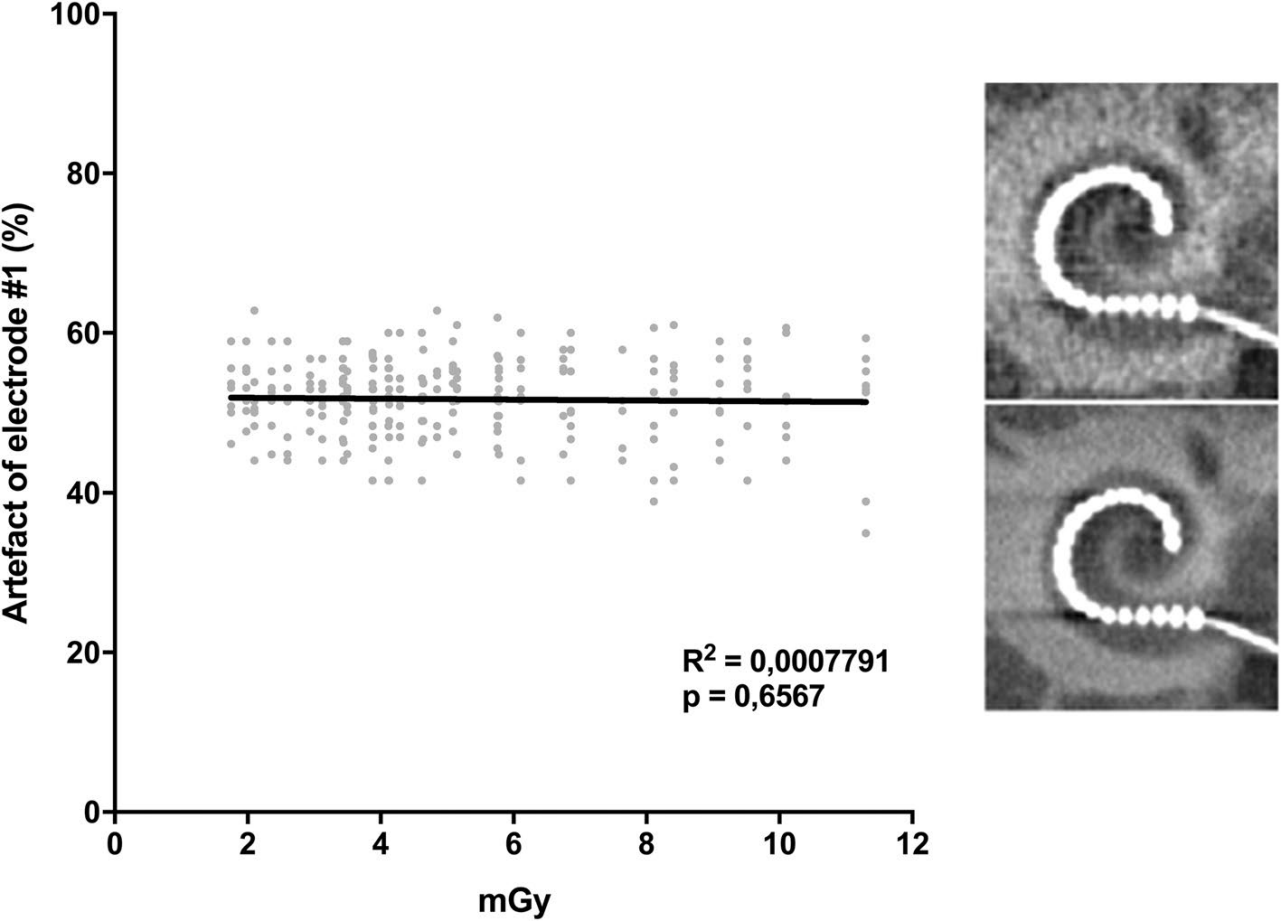
Correlation between radiation dose and artifact. The graph depicts 256 single data points. Regression (*R*^2^) and corresponding correlation (*p*). Top picture: CBCT at 1.75 mGy. Bottom picture: CBCT at 16 mGy

Since the diameter of electrode #1 did not adhere to the bony structures of the cochlea, it was used for the comparison to the applied radiation dose. The applied dose in mGy was calculated by the CBCT device and expressed in CTDI. The size of the artifact portion does not depend on the radiation dose as shown by the correlation analysis (*R*^2^ = 0.0007791; *p* = 0.66) (Fig. 4).

The measured internal diameter of the cochlea decreased significantly from base to apex, with the diameter at electrode #1 corresponding to 2.15 ± 0.14 mm and the diameter at electrode #20 corresponding to 2.03 ± 0.19 mm. Although no significant difference was found between the diameter at electrode #1 and #11 (p = 0.25 in students *t* test), the diameter at electrode #20 was significantly smaller than the diameter at electrodes #1 and #11 (*p* #1 vs. #20 < 0.001; *p* #11 vs. #20 < 0.001; students *t* test). The artifact portion was then compared with varying different exposure settings (voltage, current, rotation angle, and reconstruction mode). There was no significant correlation between the used settings and the artifact.

The Straight array follows the shape of the cochlea passively, while the Contour array is designed to adhere to the modiolus by active diffraction. The differences in distance to the outer wall of the cochlea between these arrays were significant at each electrode (*p* #1 < 0.001; *p* #11 < 0.001; *p* #20 < 0.001; students *t* test). For the Straight array, the mean distance of the artifact to the outer wall of the cochlea was 0.46 ± 0.09 mm. At electrode #11 and #20, the artifact adhered completely to the outer wall of the cochlea. The Contour array was significantly closer to the modiolus. The distance to the outer wall of the cochlea for electrode #1 is 0.8 ± 0.1 mm and narrows for electrode #11 to 0.3 ± 0.1 mm and for electrode #20 to 0.42 ± 0.07 mm.

Having confirmed that the two CI types differ in their position inside the cochlea, we investigated whether the position has an influence on the visualization of the electrode. The diameter of each electrode was measured in the coronal and sagittal plane. The average diameter measured in the coronal plane was 1.00 ± 0.16 mm at electrode #1, 1.05 ± 0.05 mm at electrode #11, and 0.95 ± 0.09 mm at electrode #20. In the sagittal plane, the average height measured at electrode #1 was 0.94 ± 0.15 mm, at electrode #11 was 1.11 ± 0.10 mm, and at electrode #20 was 1.15 ± 0.23 mm.

## Discussion

Our measurements show that the measured internal diameter of the cochlea decreases from base to the apex of the cochlea. According to Erixon et al. [17], the internal diameter of the first turn varies between 1.6 and 2.6 mm. Our radiological temporal bone measurement of the diameter was well within the described perimeter, although comparing the size of each individual cochlea is difficult, since the anatomical variance is high [18]. Compared to other radiological studies [19, 20], similar sizes of the cochlea dimensions were observed. Our methodological approach does add another factor for a high variance. As we measured the cochlea diameter at electrodes #1, #11 and #20, different CI insertion depths resulted in different absolute positions along the cochlea. In addition, the plane was centered on the electrode and not on the cochlea itself, which often leads to inaccurate representations of the diameter of the cochlea (see Fig. 1). However, the absolute dimensions are negligible, since the aim of this work was to evaluate radiological artifacts of the electrode and the dependence and the size in comparison to the applied radiation dose.

We investigated whether the radiation dose has a relevant influence on the size of the artifact. The applied radiation dose varied between 1.75 and 16 mGy. Depending on the setting the applied dose can therefore increase almost ten-fold. No dependence of the applied radiation dose on the artifact size could be found. The results suggest that a reduction of the radiation dose down to 45% of the initial value does not lead to any change in artifact size. A similar study by Weisstanner et al. [21] shows that a reduction of the radiation dose of up to 50% can also be achieved in CT without increasing the artifact size. Exact localization and display of the electrodes and the cochlear anatomy were possible even with minimization of the radiation dose. Due to the high spatial resolution and low susceptibility to metallic artifacts, the image quality remains excellent.

To compare the properties of the different arrays, the electrode position within the cochlea was determined. The Straight array passively adapted to the coils of the cochlea; while, the Contour array is designed to diffract on insertion resulting in a localization closer to the modiolus. Electrodes #11 and #20 of the Straight array are close to the outer wall and, thus, far away from the modiolus. In direct comparison, electrodes #11 and #20 of the Contour array are closer to the modiolus, resulting in a presumably better stimulation of the spiral ganglion neurons. The purpose of the Contour array is based on the premise that electrodes close to the modiolus lead to lower stimulation thresholds, and thus to improved hearing perception by increasing the spatial resolution [22]. However, insertion depth and angle do not seem to have an effect on hearing preservation [23].

In 1993, Shepherd et al. [24] described a correlation between the electrode position and the acoustic brainstem threshold in the cochlea of cats. However, Davis et al. [25] could only show a minimal contribution of localization on stimulation threshold. Further literature associates perimodiolar electrode position with increased trauma [22, 26]. This is likely caused by the significantly larger size of the Contour array compared to the Straight array, resulting in greater fluid displacement during insertion. For this reason, the Straight array is preferred for patients with residual hearing. The perimodiolar approach for electrodes was further evaluated in a recent study by Shaul et al. [27], suggesting a better hearing outcome of the CI532 compared to the CI512 with a lower of risk of translocation from scala tympani to scala vestibuli. Up to date, other studies [28, 29] could not objectify a difference in speech recognition. In the work of Shaul et al. [27], a CBCT device was used to evaluate the postoperative findings in adults, although the applied radiation dose was not mentioned. Due to the smaller size of the CI532, the round window approach is preferred for insertion [30]. It is somehow uncertain how this might have effect on the radiological presentation. Insertion trauma could be mitigated in some cases due to the use of intraoperative X-ray. It is mentioned that some trauma could not be identified by intraoperative X-ray, and was later revealed by CBCT. Intraoperative imaging gives surgeons immediate feedback, improves the definitive placement of the electrode array and opens the possibility for immediate revision surgery [4]. This is especially important due to encounter fibrosis and ossification in patients with residual hearing [2, 3]. CBCT is ideal for the visualization of CI electrodes and is becoming the reference for imaging [31].

The portion of the measured artifact compared to the real diameter of the electrode increased towards the apex of the cochlea. The Straight array resulted in an increase of more than 25% in direct comparison between electrodes at the base and at the apex, whereas the artifact portion of the Contour array only increased by approximately 15% from base to apex of the cochlea. Therefore, the localization of the individual electrode plays an important role in artifact size. With the Straight array, the cochlea wall and radiological electrode artifact are superimposed, resulting in an artificial enlargement of the respective artifact, since the exact limits of the electrode could no longer be differentiated. The localization of the electrode is nevertheless possible with minor restrictions. Other studies suggest that the portion of the artifact is about 50% of the measured radiological diameter [19, 20]. However, these results appear to be accurate only for electrode #1. As the real diameter of the electrode decreased, the portion of the artifact increased relatively. At electrode #20, the artifact portion was nearly 80% of the measured radiological diameter.

The contrast resolution of a CT depends primarily on the radiation dose influenced by voltage, current, and exposure duration. Resolution is determined by matrix and layer collimation [32]. In this work, current, voltage, and exposure duration were adjusted to determine whether these parameters have an influence on the size of the artifact. No significant correlation of the individual parameters on the size of the artifact could be determined.

This work concentrates its focus on the visualization of single electrodes in CBCT. It is limited due to its low specimen count. Due to its methodical approach with the usage of greyscale analysis of the image manipulation program, the measurements were carried out objectively—however—only by a single investigator. Clinical trials are necessary to assure the reproducibility of the findings to ensure patient safety.

In conclusion, a further reduction of radiation exposure is now possible through the discussion of the needed image quality for sufficient evaluation. The direct comparison between Straight and Contour arrays shows a difference in the intracochlear position of the electrodes. The increase of the artifact percentage of 50–70% from base to apex of the cochlea suggests that visualization of the medial and apical coils is particularly limited. Since no dependence of the artifact on the applied dose could be proven, a reduction of the radiation dose of up to 45% of the initial value in the position control of CI electrodes would be possible and should be discussed. The intensive discussion about the necessary image quality can lead to significantly higher patient safety. Next, the dose-dependent image quality should be evaluated in a clinical study.
